# The analysis of social network data: an exciting frontier for statisticians‡

**DOI:** 10.1002/sim.5630

**Published:** 2012-09-30

**Authors:** A James O'Malley

**Affiliations:** Department of Health Care Policy, Harvard Medical SchoolBoston, MA 02115-5899, U.S.A.

**Keywords:** Christakis–Fowler, dyad, network, peer effect, relationship, social influence, social selection

## Abstract

The catalyst for this paper is the recent interest in the relationship between social networks and an individual's health, which has arisen following a series of papers by Nicholas Christakis and James Fowler on person- to-person spread of health behaviors. In this issue, they provide a detailed explanation of their methods that offers insights, justifications, and responses to criticisms [1]. In this paper, we introduce some of the key statistical methods used in social network analysis and indicate where those used by Christakis and Fowler (CF) fit into the general framework. The intent is to provide the background necessary for readers to be able to make their own evaluation of the work by CF and understand the challenges of research involving social networks. We entertain possible solutions to some of the difficulties encountered in accounting for confounding effects in analyses of peer effects and provide comments on the contributions of CF. Copyright © 2012 John Wiley & Sons, Ltd.

## 1 Introduction

The study of social networks has existed since at least the 1930s in sociology 2 and related fields (e.g., psychology, anthropology). Although over time a great many methods have been developed, the computer age has enabled widespread implementation of existing methods and development of new methods for social network analysis. Recently, interest in statistical methodology for the analysis of social network data has led to more elaborate models and estimation methods. At the same time, a diverse range of applications of social network analysis have appeared, including in medicine 3–5.

Two major questions in social network analysis are as follows: (1) Do individuals' traits spread from person to person through a process of induction (also known as *social influence*, *peer effects*, or *social contagion*)? and (2) What factors affect the status and structure of relationships among a group of individuals? The questions are asymmetric in that the outcome and predictors exchange roles. In social influence analyses, the outcome is measured on an individual, and the network defines explanatory variables. In analyses of relationships, the network is the focal point, and the predictors may include variables measured on individuals. Most of the complications in social network analysis are due to the complex correlation structures arising from the inter-connectedness of individuals. An individual may influence or be influenced by multiple others, and the relationship status of one pair of individuals (dyad) may be associated with the relationship status of another dyad, even if no individuals are shared between dyads.

Medical research is centered on individual health outcomes (e.g., a comparative study of different treatments) or at least has the health of an individual in mind (e.g., an investigation to link a gene to a phenotype in humans). The study of social influence may involve the same outcome as a medical study (e.g., a health behavior), but the predictors include outcomes or covariates from other individuals (Section 3), often referred to as *alters*. Thus, although social influence models resemble regression models, they differ in that individuals may share treatments and one individual's treatment may involve another's outcome. Such *interference* between observations violates the stable-unit treatment value assumption, which requires that one individual's treatment not affect another's outcome 6 and which is generally presumed to hold in medical studies (especially in randomized trials).

Relational data are often binary (e.g., a designation of a friendship existing or not existing) with inferences about the ties linking individuals interpreted in terms of social selection. Predictors include network statistics quantifying the regularity with which particular configurations of ties occur (i.e., dependencies among network ties) and covariates such as characteristics of the units within the network. For example, transitivity—the phenomenon that ‘a friend of a friend is a friend’—implies that the tie B–C occurs more frequently when ties A–B and A–C are also present than otherwise. Studies with relational data as the dependent variable are common in sociology where the structure of society, groups, and organizations is of interest 7.

In the next section, we introduce variable definitions and notation. We then describe social influence models in Section 3 and relational (social selection) models in Section 4. The work of Christakis and Fowler (CF) primarily falls under the domain of social influence. However, the analysis of relationships or of the network itself is central to the field of social network analysis and so is equally important to describe. In addition, understanding the structure of a social network and the mechanisms of social selection may potentially be used to inform a social influence analysis, a topic addressed in Section 5. The paper concludes with a discussion and further comment on the contributions of CF in Section 6.

## 2 Definitions and notation

The fundamental entities in a social network are the individuals (e.g., individuals, organizations, or other social units) and the relationships between them. If the relationships between all eligible pairs of individuals are observed, the network is fully observed, and the data are said to be *sociocentric*. At the other extreme, if relationship status is only measured for mutually exclusive pairs of individuals, the data are *dyadic*. By measuring all relationships, sociocentric data provides more information about the influences acting on individuals in a social network than dyadic data, thereby allowing the study of multiple influences and the study of social structure 8. Herein we assume the data are sociocentric.

**Figure 1:**
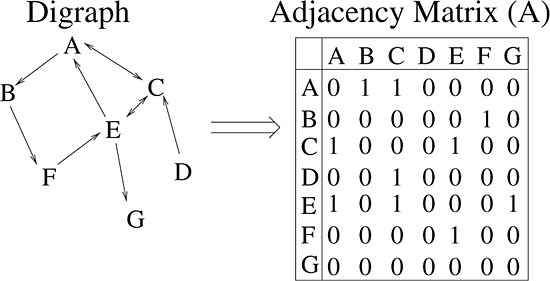
Graphical and matrix representation of a social network.

Let *y*_*it*_ and ***x***_*it*_ denote an outcome and a vector of other traits, respectively, for individual *i* = 1, …, *N* at observation period *t* = 1, …, *T* (***x***_*it*_ includes 1 as its first element to accommodate an intercept). In addition, *a*_*ij*_ denotes the relationship between individuals *i* and *j*, assumed for now to be time invariant. For ease of notation, we make no distinction between random variables and realizations of them. The vector ***Y***_*t*_ and the matrices ***X***_*t*_ and ***A*** are the respective network-wide quantities. We depict the representation of these variables in Figure 1.

In a directed network, the status of the relationship from *i* to *j* can differ from that from *j* to *i*, whereas in a non-directed network, *a*_*ij*_ = *a*_*ji*_, implying ***A*** = ***A***^*T*^. A network constructed from friendship nominations is likely to be directed, whereas a network of coworkers is non-directed. In the case of non-mutable relationships, ***A*** will only change as individuals are added or removed, as relationship status is otherwise invariant.

The out-degree and in-degree, given by 
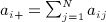
 (row sum) and 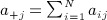 (column sum), respectively, are the number of individuals *i* (the *ego*) nominates and the number of other individuals (*alters*) nominating the ego. These are referred to as *expansiveness* and *popularity*, respectively. The degree distributions for a network reflects the heterogeneity in the numbers of ties across individuals. A positive correlation between out-degree and in-degree suggests that popular individuals are expansive—a phenomenon referred to as *homophily on degree*.

**Figure 2:**
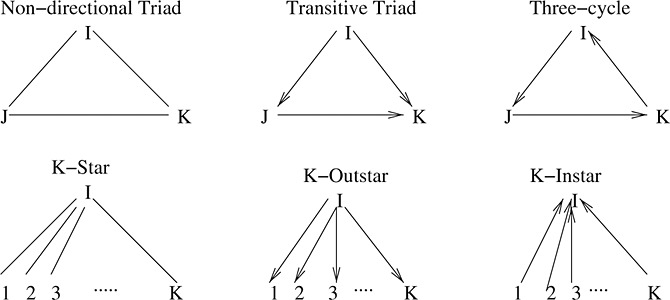
Triadic and *k*-star configurations.

Certain subnetworks have particular theoretical prominence. A pair of individuals is a *dyad*, and a triple is a *triad*. The configurations in Figure 2 are of triads and *k*-stars. A *k*-star consists of an individual and any *k* relationships incident to it. In an undirected network there is a single triad and *k*-star (for fixed *k*) configuration, whereas with directed network data a number of different configurations exist. For example, there are 16 distinct triad isomorphic classes 9, page 566. The transitive triad, three-cycle, and the *k*-outstar and *k*-instar are some of the more common configurations involving multiple dyads.

The length of a path between two individuals through the network equals the number of ties traversed. The elements of *A*^*k*^ equal the number of paths of length *k* between any two individuals; the number of *k*-cycles (including multiple loops) is on the diagonal. The shortest path between two individuals is referred to as the *geodesic distance*.

The second-degree and third-degree alters to whom an individual is connected are identified by the non-zero elements in *A*^2^ and *A*^3^, respectively. The alters that are uniquely second-degree and uniquely third-degree are those that are not connected at a lower degree. For example, because of the two-path *D* → *C* → *A* and the absence of a *D* → *A* tie, individual A is a second-degree alter of individual D. Similarly, individual G is a third-degree alter of individuals A, B, and D as there is at least one three-path but no direct tie or two-path from A, B, or D to G.

**Figure 3:**
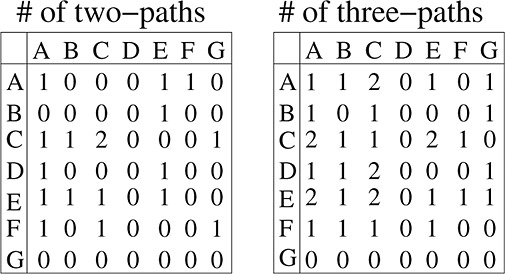
Square and cube of adjacency matrix. The numbers in the cell give the number of paths of lengths 2 (left matrix) and 3 (right matrix). Cell counts > 0 indicate whether a second-degree or third-degree relationship exists from one individual to another (the diagonal elements indicate ‘self-relationships’).

The importance of second and higher-order ties in social networks is a topic that has recently been debated because of the claim by CF that peer influence extends to three degrees of separation 10. For simplicity, suppose for the moment that only individuals who are named as friends impart influence on the nominating individuals. In the context of the digraph in Figure 1, whose two-paths and three-paths are indicated in Figure 3, an example of second-degree influence is the effect of A on D above and beyond that of C on D. Because the only two-path from D to A is through C, the (second-degree) effect of A on D corresponds to the outcome for D under the given network less the counterfactual outcome for D if the C to A tie was removed (i.e., the only change to the network is the removal of the two-path from D to A). An example of third-degree influence is the influence of G on A above and beyond the total effects of the first-degree and second-degree alters of A. Because the only three-path from A to G is though C and then E, the only way in which the three-path is broken without altering the direct ties and second-degree paths emanating from A is by removing the tie E–G. Therefore, the (third-degree) effect of G on A equals the outcome for A under the given network less the outcome for A if counterfactually the tie E–G was removed.

**Figure 4:**
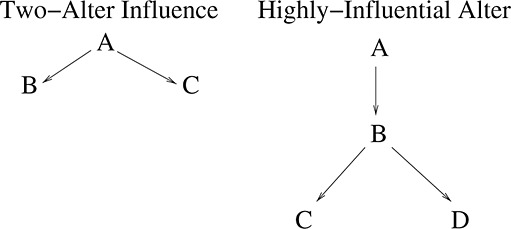
Graphical and matrix representation of a social network.

It is important to note that the effects described in the preceding paragraph are specific to pairs of individuals. For identifiability, the effect of second-degree, third-degree, or *k*-degree influence in a network must be defined with respect to a model that describes how influence acts in the network under various scenarios. For example, individuals in different positions of the network might have different numbers of direct and higher-order ties and receptivity to thresholds. For example, two or more direct ties to individuals with a given behavior state might be needed in order for an individual to alter their own behavior (e.g., individual A in the left-side network in Figure 4 is susceptible to combined influence from B and C). Alternatively, one direct tie might be sufficient if the alter has two or more direct ties to other individuals, making the alter more persuasive than if they had one or no ties to other individuals (e.g., individual A in the right-side network in Figure 4 is susceptible to influence from B if B's exposure to C and D makes B very influential.

Clearly, there are a plethora of possibilities that a model of influence in a social network may seek to represent. The network influence model described in Section 3 assumes a simple mechanism for influence in the network and does not account for the types of modifying effects discussed earlier. The development of more elaborate models (or statistical tests) is an open area for further research.

## 3 Network influence models

Regression models for estimating peer effects are primarily concerned with how the distribution of some dependent variable (e.g., a behavior, attitude or opinion) measured on a focal individual is related to one or more explanatory variables. When behaviors, attitudes, or opinions are formed in part as the result of interpersonal influence, outcomes for different individuals may be statistically dependent. The outcome for one individual will be related to those for the other individuals who influence her or him, leading to a complex correlation structure.

In social influence analyses the weight matrix, ***W*** = [*w*_*ij*_], apportions the total influence acting on an individual across the other individuals in the network. Typically,

1. *w*_*ij*_ *≥*0: non-negative weights.
2. *w*_*ii*_ = 0: no self-influence.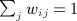: weights give relative influences (***W*** is row-stochastic).

Then 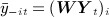 denotes the influence-weighted average of the outcome across the network excluding individual *i*, and 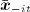 denotes the vector containing the corresponding average covariates, often referred to as *contextual variables*.

**Figure 5:**
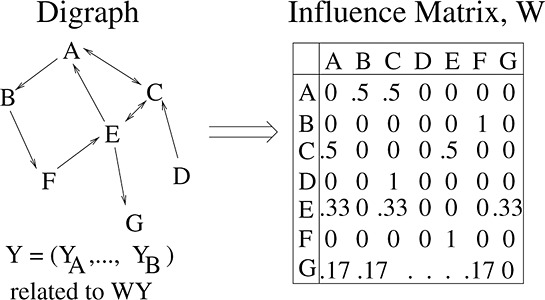
Construction of network influence matrix.

In general, ***W*** is derived from ***A***, such as the row-stochastic version of ***A***. We consider the case where ***A*** is binary (the elements are 1 for tie and 0 for no tie). Thus, the non-zero elements on the *i*th row of ***W*** equal 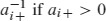 and 1/(N − 1) otherwise (see Figure 5). This framework assumes that an individual's alters are equally influential.

We may incorporate network-related interdependence among the outcomes in two distinct ways. First, an outcome for one individual may depend directly on the outcomes of the alters to whom he/she is linked. For example, consider the discrete-time dynamical system (Markov transition model):

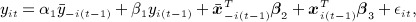
(1)
where *α*_1_ is a scalar parameter quantifying the peer effect; 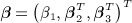 is a vector of regression parameters, and *ε*_*it*_ is the independent error assumed to have mean 0 and variance *σ*^2^. Equation 1 is known as the ‘linear-in-means model’ 11. We obtain a number of commonly used variants of this model by adding or omitting predictors from Equation 1.

If there are multiple types of alters, we can use network influence models with multiple influence matrices:

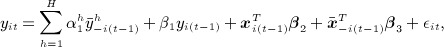
(2)
where 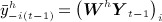, and *W*^*h*^ denotes the influence matrix for relationship type *h* (influence is 0 if a given tie is not relationship type *h*). In the special case where 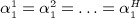, 2 reduces to 1.

**Figure 6:**
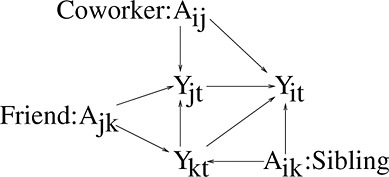
Directed acyclic graph: potential confounding effects of multiple peers.

Alternatively, separate models can be fit for each type of peer. However, failing to simultaneously account for all alters may lead to biased results. For example, in Figure 6 individual *k* influences individuals *i* and *j* and so is a confounder of the effect of *i* on *j* and the effect of *j* on *i*. Ignoring individual *k* may lead to biased estimates of the coworker peer effect.

Clear boundaries or rules of inclusion for units/actors must be specified to define the network 12. However, in situations where the boundaries break true ties, influential peers may be excluded, potentially leading to biased results.

### 3.1 Estimation of contemporaneous peer effects

From a practical standpoint, it may be infeasible to use a model with only lagged predictors such as Equation 1. For instance, the time points might be so far apart that statistical power is severely compromised. Therefore, it is tempting to use a model with contemporaneous predictors. For example, the network influence model comparable with the model fit by CF is given by

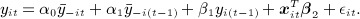
(3)
However, inclusion of 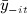 in 1 as a predictor leads to simultaneous feedback and endogeneity. Because the predictors are correlated with the outcomes of other equations, ordinary least squares will be inconsistent. Therefore, methods to account for endogenous feedback are needed.

A parametric model-based solution to the problem of endogenous feedback follows from specifying a joint distribution for ***ε***_*t*_ = (*ε*_1*t*_, …, *ε*_*Nt*_). Then obtain the reduced form of the model by solving ***Y***_*t*_ = *α*_0_***WY***_*t*_ + *α*_1_***WY***_*t* − 1_ + *β*_1_***Y***_*t* − 1_ + ***X***_*t* − 1_***β***_2_ + ***ε***_*t*_ for ***Y***_*t*_ to yield ***Y***_*t*_ = (***I*** − *α*_0_***W***)^ − 1^{*α*_1_***WY***_*t* − 1_ + *β*_1_***Y***_*t* − 1_ + ***X***_*t* − 1_***β***_2_ + ***ε***_*t*_}.

The preceding model is analogous to a spatial autoregressive model, a family of models that have been used extensively in the field of spatial econometrics 13. The traditional problem to which these models have been applied is the estimation of the effect of the aggregate or average level of a variable in neighboring areas on the same or a different variable in the focal area. Furthermore, we may define the elements of ***W*** on the basis of indicators of whether areas are neighbors (as in areal data) or some measure of distance between areas. Thus, the problem-type and representation of ***W*** in spatial econometrics and network influence may be similar.

Despite the similarities, there are several ways in which analysis of network influence involves complexities not encountered in the traditional spatial econometric settings. First, influence in networks can be directional, whereas adjacency matrices for areas are symmetric. Second, because of the absence of an underlying ‘physical distance’, the topology of the network is generally more complex than under models for spatial correlation. Third, because areas do not select other areas to be neighbors, the notion of homophily does not apply to spatial econometrics. Thus, although the general model resembles those in spatial econometrics, network influence analysis encounters several additional challenges.

Concerns about spatial econometric models arise because identification of the model relies on the truth of the assumed distribution of ***ε***_*t*_, which cannot be empirically tested from the data. Therefore, results are likely to be sensitive to departures of the distribution of ***ε***_*t*_ from normality, especially when the peer effect is contemporaneous. This concern echoes those expressed towards bivariate probit models that simultaneously model a system of equations comprising the outcome given the treatment, the treatment given the selection variables, and unmeasured confounding represented in terms of the correlation between underlying latent residuals 14.

### 3.2 Causal estimation

An alternative to using a full parametric model to account for endogeneity is an instrumental variables analysis (CF refer to work underway on this approach). In the context of contemporaneous peer influence, an instrumental variable (IV), *z*_*i*_, must be correlated with 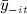 conditional on other all other observed and unobserved predictors of *y*_*it*_ but not be correlated with *y*_*it*_ conditional on 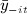 and any other observed predictors of *y*_*it*_. However, IV methods can be problematic if the instrument is weak or if the assumption that the IV does not affect *y*_*it*_ through any unblocked pathways is violated. The latter is known as the *exclusion restriction* and is itself an untestable assumption. Thus, in fitting a model with contemporaneous peer effects, alternative identification strategies exist: make a multivariate parametric assumption, assume the non-existence of unmeasured confounding variables, or assume that an instrumental variable is valid. In general, none of these assumptions can be conclusively evaluated using the observed data.

In general, finding a valid instrument is a difficult task. However, it is more difficult in the context of peer effects as there are multiple types of unmeasured confounders, and in order to satisfy the exclusion restriction, an IV must not be causally associated with any unmeasured confounders. In a peer effects analysis, the analogy of a confounder of treatment in a medical study (i.e., a variable that affects both the outcome and the treatment) is a variable that simultaneously affects the outcomes of multiple individuals, often referred to as a ‘common cause’. An unmeasured common cause is thus an unmeasured confounder in the context of peer effects. However, a second and more subtle form of confounding arises whenever factors affecting an individual's propensity to form or break 15 tie(s) with other individuals also affect the outcome measured on that individual. The former occurs whenever similarity (dissimilarity) on a trait make two individuals more likely to form (break) ties, a process known as homophily. Because relationship status is conditioned on in a peer effects analysis, a variable that induces homophily is correlated across connected individuals 16. If that variable affects the outcome variable, then it is correlated with the outcomes of the other individual comprising the dyad. Unmeasured variables that lead to homophily and also affect the outcome are thus indirect common causes. Therefore, the exclusion restriction in a peer effects analysis requires that the IV is uncorrelated with unmeasured common causes of the outcome and unmeasured sources of homophily that affect the outcome.

Because the contextual variables 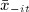 and 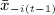 are excluded from equation 3, we can potentially use their elements as IVs for (contemporaneous and lagged) peer effects 17. However, in practice, it is important to assess whether any candidate IVs are associated with tie-formation or tie-dissolution. A variable that contributes to homophily will be correlated with any unmeasured confounders and therefore would not be a valid IV.

### 3.3 Dyadic influence model of Christakis and Fowler

Christakis and Fowler apply a model designed for dyadic data (each pair of individuals is mutually exclusive) to longitudinal sociocentric data constructed in a novel way to data from the Framingham Heart Study (FHS) offspring cohort and data from other longitudinal social network studies (e.g., Add Health). As opposed to using individual exams as the units of analysis, this approach uses ego's observation at exam *t* as an outcome for each ego–alter pair that remained intact from exam *t* − 1 2012, Section 4. Thus, the predictors are based on an individual alter as opposed to representing the net influence across all alters of the focal individual. If ***W*** is time invariant, the analysis dataset consists of *L ≥ N* ‘observations’ at *t*, where *L* is the number of positive elements in ***W*** when only *N* values of the outcome are measured at any given time. The observation distortion only dissolves when each dyad contains disjoint pairs of individuals, in which case *L* = *N*.

We obtain the dyadic model analogous to 3 by replacing − *i* with *j*; that is, substitute 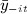 with 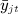 to obtain the following:

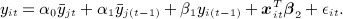
(4)
Christakis and Fowler estimate the model parameters in 4 using generalized estimating equations (GEE). Thus, they avoid specifying a distribution for *ε*_*it*_. Because the dependent variable is repeated across observations for individuals with multiple alters, fitting a dyadic influence model on sociocentric data has some similarity with an analysis in which a predictor but not the outcome is evaluated by multiple informants 18. However, social network data is more complicated because an ego (individual *i* in 4) can be an alter for other egos (potentially any individual *k* ≠ *i*). Because of the fact that the GEE procedures available in statistical packages do not account for the statistical dependence introduced by individuals who play the dual role of ego and alter at the same *t* 19, we need specially developed methods to ensure that inferences are valid.

Marsden and Friedkin 20 previously discussed the merits of the network and dyadic influence approaches. They note that if one assumes that the dyads are disjoint when a person is, in fact, influenced by multiple others (as in Figure 6), then estimates of peer effects may be biased. Citing 1991, they suggest that such bias is likely to be downward. Thus, the dyadic influence approach of CF is exposed to bias from the confounding effects of other peers. However, it is also important to note that in several CF analyses of the FHS network data, FHS study members predominantly have a single alter (e.g., an ego or alter friend 1), in which case the network and dyadic influence approaches are equivalent. More work is needed to assess the relative benefits in terms of bias and robustness to model misspecification of the network and dyadic influence models for sociocentric data.

## 4 Relational analyses

In Section 3.2, we described the problems posed by homophily to the identification of causal peer effects. In order to assess whether homophily exists and, if so, estimate the size of its effect, relationship status may be regressed on measures of homophily. If the estimated coefficients of the homophily effects are close to 0, then one might feel more secure with the estimates obtained from a peer effects analysis. Furthermore, we may use estimated homophily effects as inputs to specify the magnitude of the effect of an unmeasured confounder in a sensitivity analysis of estimates of peer effects 16. However, as described in the remainder of this section, modeling relational data is thwart with its own set of challenges.

Because sociocentric data are inter-connected, the relationships in a social network must be modeled simultaneously. Models for such data posit that global network properties are the result of a set of localized regularities that create correlations involving subsets of network ties, for example, within individuals, dyads, triads, or tetrads 22. Examples of such regularities are individual-level tendencies to produce and/or attract ties, dyadic tendencies toward reciprocity, and triadic tendencies toward closure or transitivity. A relational model, in essence, specifies a set of micro-level rules governing the local structure of a network.

Relational models may also incorporate attribute data on individuals or relationships. For instance, certain types of individuals may tend to attract ties, individuals having the same or similar attributes may tend to be linked (homophily), or individuals linked at one point in time may tend to be connected in networks at subsequent times. This point is relevant to network influence models. A model that correctly describes sociocentric data must account for all sources of homophily.

The simplest models for sociocentric data assume dyadic independence. Under the constant or completely random model, all ties have equal probability of occurring, and their status is independent of each other 23. Models with dependence between the ties within the dyad but independence between dyads constitute the next most sophisticated form of model. In directed networks, the first dyadic models were developed by Holland and Leinhardt 1981 and later were extended by Wang and Wong 1987. These have the form of a regular statistical model in that the likelihood function is the product of contributions from the dyadic observations:

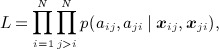

where for ease of depiction we treat ***x***_*ij*_ as time invariant and note that it may include *y*_*i*_ and *y*_*j*_ (the trait of interest from the network influence models). We discuss longitudinal extensions and joint models in Section 5.

In cases where the relationship states of different dyads are independent, a four-component multinomial distribution for (*a*_*ij*_,*a*_*ji*_) determines the model for the network. The dyadic state probabilities are typically represented in the form of a generalized logistic regression model, such as

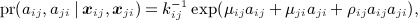
(5)
where

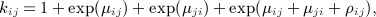

and *μ*_*ij*_, *μ*_*ji*_ and *ρ*_*ij*_ are functions of (***x***_*ij*_,***x***_*ji*_) and associated parameters. The term *μ*_*ij*_ includes factors associated with the likelihood that *a*_*ij*_ = 1 but not necessarily the likelihood that *a*_*ji*_ = 1. The term *ρ*_*ijt*_ = *ρ*_*jit*_ includes factors that affect the correlation of *a*_*ij*_ and *a*_*ji*_ (mutuality). These allow the probability of *a*_*ij*_ = *a*_*ji*_ = 1 to deviate from exp(*μ*_*ij*_ + *μ*_*ji*_) / *k*_*ij*_, the probability obtained under independence of *a*_*ij*_ and *a*_*ji*_ (in which case, *ρ*_*ij*_ = 0).

The terms *μ*_*ij*_, *μ*_*ji*_, and *ρ*_*ij*_ in 6 can include network-based covariates that are specific to the dyad such as the elements of the dyad census (out-degree, in-degree, number of mutual ties). Effects can be homogeneous across individuals or individual-specific. For example, the p _1_ model 24 assumes *μ*_*ij*_ = *μ* + *α*_*i*_ + *γ*_*j*_ and *ρ*_*ij*_ = *ρ*, implying the joint probability density function of the network given by

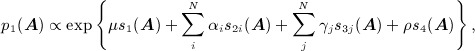

where 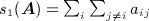, 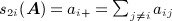, 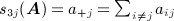, and 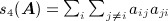. Thus, the p _1_ model depends on 2*N* + 2 network statistics. If the p _1_ model holds within (ego, alter)-shared values of categorical attributes (i.e., within blocks), the model has the form of a stochastic block model 26,27. Stochastic block models are the basis of mixed-membership and other recent statistical approaches to detection of clusters (termed ‘communities’) in social network data 28,29; this is an important topic with a growing number of applications but is beyond the scope of this paper.

### 4.1 Models of networks as single observations

A criticism of dyadic independence models is that they fail to account for interdependencies between dyads. If such effects exist, then the effects of other variables in the model are susceptible to omitted variable bias. The p* model or exponential random graph model (ERGM) generalizes dyadic independence models to a much more flexible model 30,31. An ERGM has the general form

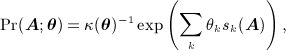
(6)
where 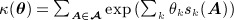 and 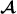 is the set of all 2^*N*(*N* − 1)^ networks.

Under dyadic dependent ERGMs, the network is represented by a categorical random variable with 2^*N*(*N* − 1)^ categories that in general cannot be simplified. To illustrate, we show that when the data are sociocentric, the unit of analysis for a triadic model is in general the whole network. A triadic model contains no interactions of four or more ties and is commonly referred to as a *Markov random graph* 30. Because triads are considered an important social unit—closed triads are thought to reinforce/stabilize ties—such a model would be of great interest. In a binary-valued network, a triad has 2^3^ = 8 possible states, and a network contains *N*(*N* − 1)(*N* − 2) / 6 triadic observations. However, unlike dyads, the state of one triad places a constraint on the possible states of a triad with any two individuals in common. Thus, triads are not mutually exclusive units. As a consequence, the appropriate analytic unit in a triadic (or higher-order) model is the whole network. Then the scale factor *κ*(***θ***), a sum over each distinct network, does not factor into a product of analogous terms. This makes it computationally infeasible to exactly evaluate the likelihood function of dyadic dependent ERGMs for *N* much greater than 20 32.

An attractive feature of ERGMs is their flexibility in allowing a wide range of hypotheses and sociological constructs to be tested through the inclusion of the appropriate network statistics. The conditional likelihood of each tie given the rest has the following logistic form:

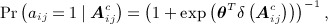
(7)
where 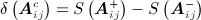 is the vector of changes in network statistics that occur if *a*_*ij*_ is 1 rather than 0, and 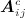 is ***A*** absent *a*_*ij*_. Thus, parameters reflect the change in the log-odds that the tie is present, conditioned on the rest of the network 33.

Equation 7 gives rise to two often-used estimation methods for ERGMs. ERGMs were first estimated using a pseudolikelihood function defined as the product of the conditional distributions implied by 10 over ordered pairs (for directed networks) or ties (in the undirected case) 31,34. Because the pseudolikelihood has the same form as a logistic regression likelihood function, parameter estimates are easily obtained. However, unless the model is dyadic independent, the pseudolikelihood differs from the true likelihood function, and estimates may not be consistent 35.

We can improve upon pseudolikelihood estimates by using numerical methods to approximate the exact likelihood function for 9. Recently developed Markov chain Monte Carlo methods allow inferences to be based on the true likelihood function. The R package statnet 36, which can fit models to moderately sized networks (up to thousands of individuals 37), implements this approach. Getting estimation procedures for ERGMs to converge can be difficult because the likelihood surface implied by 9 often has a highly irregular shape, resulting in algorithms becoming trapped at local maxima, failing to converge, or converging to inappropriate degenerate solutions. The latter problem, known as *degeneracy*, arises because for certain specifications of *s*_*k*_(***A***) there may be few realized networks with positive probability; such networks may be radically different from each other (e.g., the empty and the complete networks), the network statistics defining the model are highly correlated, and the likelihood function has multiple local optima. As a consequence, randomly generated networks under fitted ERGMs may yield samples of networks, none of which remotely resembles the observed network 36,38.

Although ERGMs have been fit to networks with over a thousand individuals, in general the feasibility and reliability of model estimation is sensitive to the network statistics that define the model. For example, the inclusion of the number of triangles (directed or otherwise) can be particularly problematic as fitted models are often degenerative. This has led to the development of new specifications of common statistics such as triangles and *k*-stars. For example, in place of a 3-star and a 4-star, a single statistic corresponding to an alternating sum of *k*-stars is used. A similar generalization has been developed for *k*-triangles; it corresponds to a weighted sum of the number of shared partners of each individual in the network 37.

### 4.2 Conditional independence approaches

Alternative approaches to ERGMs have utilized random effects to avert some of the computational problems associated with ERGMs. An example is the mixed effects p _2_ model in which the expansiveness *α*_*i*_ and popularity *γ*_*j*_ parameters under the p _1_ model are instead treated as a random sample from a distribution whose parameters are to be estimated 39,40. In the p _2_ model, (*α*_*i*_,*γ*_*i*_) is typically assumed to be bivariate normal. The p _2_ model also accommodates individual and dyadic covariates—covariates reflecting some feature of the dyad (e.g., both smokers, both older than 65 years). The p _2_ model is given by

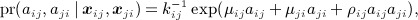
(8)(8)

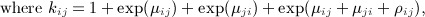


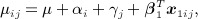


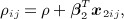


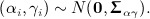


Thus, conditional on (*α*_*i*_,*γ*_*i*_,*α*_*j*_,*γ*_*j*_), the relationship status of dyad *ij* does not depend on that of another dyad. A positive off-diagonal element of ***Σ***_*αγ*_ implies homophily by degree (expansive individuals are popular).

Recently, a number of models that use latent variables to account for between-dyad dependence have been developed. That is, the observed relational data are determined in part by unobserved latent variables that might be shared or correlated between individuals. The major types of models are latent class models 41,42, latent space or distance models 43,44, and latent eigen(-factor) models 45,46. Technically, these models are conditional tie-independence models as they are either designed for undirected networks or model reciprocity using latent variables 45. An alternative is to extend the p _2_ model, which represents reciprocity as an interaction between observed variables rather than as a correlation between latent variables, analogously to the models in 46 by augmenting *μ*_*ij*_ or *ρ*_*ij*_ with either the following:

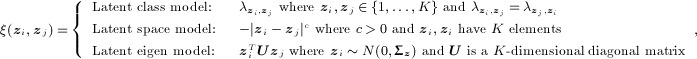
(9)
respectively.

In the latent class model, the array of values of 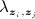 forms a symmetric *K* × *K* matrix ***Λ***. A basic specification is 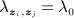 if ***z***_*i*_ = ***z***_*j*_ (nodes in same partition) and 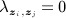 if ***z***_*i*_ ≠ ***z***_*j*_ 42. Latent class models extend stochastic block models to allow the blocks to be latent (estimated from the data) as opposed to user-specified. This family of models is well suited to network data thought to be clustered, as might occur if there existed underlying (i.e., unobserved) communities or other groups within which observations were considered structurally equivalent.

In the latent space model, the most common values for *c* are 1 and 2, corresponding to absolute and Cartesian distance, respectively. For example, 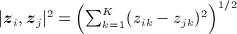, where *K* is the dimension of the latent space. The distance metric accounts for latent homophily—the effect of unobserved individual characteristics that induce ties between individuals. In this model, ***z***_*i*_ represents individual *i*'s unobserved latent position in a social space 43,47. The model accounts for triadic dependence (e.g., transitivity) by requiring that latent distances between individuals obey the triangle inequality. Latent space models are available in the latentnet package in R 44.

The latent eigen model is the most general specification and accounts for both latent clustering and homophily. Furthermore, the parameter space of the latent eigen model of dimension *K* generalizes that of the latent class model of the same dimension and weakly generalizes the latent distance model of dimension *K* − 1. Conversely, the latent distance model of dimension *K* does not generalize the one-dimensional latent eigen model 46. The term 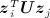 captures transitivity by constraining the extent to which the inner products 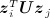, 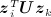, and 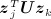 can vary from one another. Specifically, the likelihood of a tie between *i* and *j* will increase if ***U***^1 / 2^***z***_*i*_ and ***U***^1 / 2^***z***_*j*_ have a similar direction and magnitude, allowing for more clustering than under 11. The greater the magnitude of ***Σ_*z*_***, the greater the extent to which ties are expected to cluster and closed triads will form.

The challenges with models involving latent variables resemble those in factor analysis or other dimension reduction models. For one, determining the value of *d* may not be straightforward. Second, computational challenges in estimating the latent variables can make the method difficult to apply to larger networks. Nonetheless, a great virtue of this approach is that the problem of degeneracy is avoided, and furthermore, solutions are almost always well defined. However, these models do not suffice if one is interested in testing hypotheses about specific higher-order effects (e.g., separating the effect of transitivity from three-cycles or higher-order forms of closure) as the effects are not distinguishable. However, if longitudinal data are available, higher-order configurations can enter the model as lagged predictors 48.

Although models using latent variables to account for inter-dyad dependence are restrictive as they do not distinguish between higher-order effects, they are generative in the sense that the model for a dyad determines the distribution of the network. Therefore, the joint model of the network can be expanded into conditional distributions of each dyad and marginal distributions for the random effects. Because the likelihood function is the product of analogous terms evaluated on each dyad, regular asymptotic and other theoretical results apply, and degeneracy is a non-issue.

Another perspective of the difference between ERGMs and (conditional) dyadic independence models is that ERGMs are defined through the specification of the sufficient statistics of the network. Thus, the model is specific to the observed network and cannot be used to generate a network with different features (e.g., a different number of individuals). In contrast, dyadic conditional independence models that involve latent variables emulate regular statistical models by describing the population from which dyads (and thus networks) are drawn. Therefore, the model can be used to make predictions about networks with a different number of individuals, density of ties, or values of other predictors.

## 5 Advanced topics

### 5.1 Longitudinal relational models

Although the causal basis of network influence models makes the use of longitudinal data highly desirable, the development of relational models has primarily focused on cross-sectional data. Longitudinal variants of ERGMs have only recently been developed. Extensions of ERGMs to the discrete Markov domain have been developed by Hanneke and colleagues 49,50 and Krivitsky and Handcock 2010. The first longitudinal ERGM-type models treated tie-formation and tie-dissolution as equitable events in the evolution of the network 49,50. A more general formulation treated tie-formation and tie-dissolution (the complement of tie duration) as separable processes, thereby allowing the same network statistic to impact tie-formation and tie-dissolution differently 51.

Like ERGMs for cross-sectional data, the preceding longitudinal models are defined by statistics that count the number of occurrences of substructures in the network. However, in addition to the current state of the network, such statistics may also depend on previous states. Under Markovian dependence, network statistics only depend on the current and the most recent state. For example, the number of ties that remain intact from the preceding observation is accommodated in a Markov transition model.

An alternative approach for modeling network evolution is the actor-oriented model 52–54. This centers on an objective function for individuals that may be sensitive to multiple network properties including reciprocity, closure, homophily, or contact with prestigious others. The model assumes that individuals control their outgoing ties and change them in order to increase their satisfaction with the network in one or more respects. It resembles an economic model of rationale choice in which each individual attempts to maximize their own utility function. These models combine a continuous time process that controls the opportunity of change with a discrete propensity of change based on a utility function. Estimated parameters indicate whether changes in a given property raise or lower individual satisfaction.

An important distinction of actor-oriented models from ERGMs is that the relevant network statistics in the actor-oriented model are specific to individuals rather than being aggregations across the network. Because these actor-oriented models resemble the ERGMs in the limiting case, they also suffer from degeneracy, although the problem is less profound as it occurs in the limit 55. Furthermore, like ERGMs it is computationally intensive. The siena package in stocnet 56,57 uses a stochastic approximation algorithm for estimation that allows flexibility in the form of missing tie-level data but is most feasible when applied to relatively small networks.

Longitudinal counterparts of (latent) conditional independence models have also been developed that use either fixed or random effects to account for dependence over time. The model is extended by adding terms that account for longitudinal dependence (e.g., past states of the dyad) and an index *t* for observation number. A simple case of such a model was developed by O'Malley and Christakis 2011. The Markov transition model they use assumes that tie-formation and tie-dissolution are unrelated processes and that, conditional on the past state of the dyad and the sender and receiver random effects, ties are statistically independent random variables. A more general formulation is the full longitudinal extension of the p _2_ model, which allows within-dyad tie-dependence (reciprocity), homogeneous or heterogeneous effects between formation and dissolution of ties, and the inclusion of higher-order effects (e.g., third-order interactions such as transitive triads) as lagged predictors 48. A further extension is a longitudinal latent space model that accounts for third and higher-order contemporaneous interactions in tie-states. Such a model has also been entertained by Westveld and Hoff 2011.

### 5.2 Joint influence-relationship models

A virtue of the actor-oriented modeling framework in siena is that effects related to individual's relationships (social selection) can be modeled jointly with the effects of an individual's peers (e.g., a friend or a neighbor) on their own traits (social influence). Such a model was developed by Steglich and colleagues 2010.

An alternative approach that is closer to traditional statistical models for joint outcomes is to jointly model influence and selection using shared latent variables. The rationale underlying this approach is that unmeasured factors that simultaneously affect both social influence and social selection are captured by the latent variables. The same rationale applies to joint models used for survival and longitudinal outcomes. In the case of social networks, if the joint model is correctly specified, then it accounts for unmeasured homophily (a concern in the analyses of CF) and other confounding effects enabling consistent estimation of the effect due to social influence. For example, a joint model constructed from the social influence model in 1 and the p _2_ relational model in 11 but without reciprocity covariates has the following form:

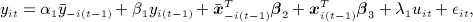
(10)

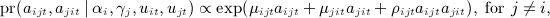
(11)

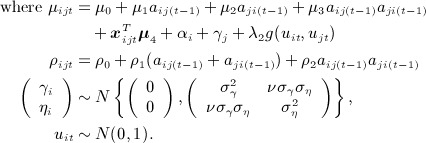
(12)
An example of an additive and a multiplicative specification of the latent variables are *g*(*u*_*it*_,*u*_*jt*_) = *u*_*it*_ + *u*_*jt*_ and *g*(*u*_*it*_,*u*_*jt*_) = *u*_*it*_*u*_*jt*_, respectively.

The presence of *u*_*it*_ and *u*_*jt*_ in 10 and 11 captures unmeasured factors affecting social influence and social selection (e.g., homophily) thereby accounting for latent homophily. The extent of the correlation is quantified by the coefficients *λ*_1_ and *λ*_2_. Although statistically efficient, joint models are often sensitive to model misspecification. Beyond the separate assumptions of each model, the validity of results under 10–12 relies on the closeness of the distributions assumed for ***ε***_*t*_ and *u*_*it*_ to the true distributions and, similarly, the closeness of *g*(*u*_*it*_,*u*_*jt*_) to the true specification.

To the author's knowledge, a model like 17–19 has not yet been developed. Several issues need to be resolved before such a model could be used in practice. These include the extent to which constraints on *λ*_1_ and *λ*_2_ are needed in order for the model to be identifiable by the data but without unnaturally restricting the magnitude and sign of the correlation between (*y*_*ijt*_,*y*_*jit*_) and (*a*_*it*_,*a*_*jt*_).

## 6 Discussion

This paper has introduced the components of social networks and statistical models for analyzing complete network (i.e., sociocentric) data. We have described methods for evaluating whether individuals' attributes spread from person to person across a population (social influence), the hypothesis at the forefront of the CF papers, and for modeling relationships in social networks. In addition, we proposed a joint influence-selection model as a parametric alternative to instrumental variable analysis in identifying causal effects of social influence (distinct from latent homophily and other confounding factors) under the assumption that the model is correct. While prioritizing methods of most relevance to the work of CF, we have not discussed several important topics in social network analysis. These include several descriptive measures of networks (see 9 for a thorough review), analysis of bipartite networks, community detection models and algorithms, egocentric network analysis, visualization of networks, and numerous other topics.

It is often said that 99% of the work in statistics is acquiring the data and preparing it for analysis. In the FHS Network dataset, CF have developed a unique resource for which they deserve substantial credit. By forging ahead and producing important results despite rock-solid statistical techniques not being implementable (or even available), they have raised the profile of social network analysis and been the catalyst to an informative debate on methods for social influence. A strength of the FHS is the reliably measured data on individuals' health and physical measurements. Although the internet and electronic media (e.g., cell phones, Twitter) have expanded the capability of researchers to form networks, attribute information is often obtained through self-report without any confirmation of its accuracy. Therefore, data such as the FHS network have the potential to be a valuable resource for several years into the future.

The tie-directionality identification strategy developed by CF to account for confounding due to unmeasured common causes 1, Section 5 is a novel idea based on sound intuition. Although their procedure does not guard against all sources of unmeasured confounding (in general, this is impossible in an observational study), it accounts for many sources of them. Because it is based on a solid theory, the directionality test provides a stronger form of evidence than a generic sensitivity analysis. That said, we always recommend accompanying the directionality test with alternative models involving only lagged predictors or appropriate sensitivity analyses.

The field of social networks is growing rapidly in methodological development and applications. Furthermore, a parallel field called *network science* exists that comprises physicists, computer scientists, engineers, and mathematicians (and various other disciplines). In the course of their research, CF have used or adapted several techniques from network science. One example is their use of permutation tests to estimate the degree of separation to which social clustering can be detected 61. Their permutation test randomly re-assigns the trait of interest across the network, performs the analysis of interest, and iterates between these two steps multiple times to obtain a null distribution for evaluating significance levels 1, Section 2. However, the null hypothesis of no clustering whatsoever is not the null hypothesis of primary interest. A claim that clustering (on obesity or smoking) extends to three degrees would be more convincing if the next simplest case (i.e., clustering to two degrees) was the model under the null hypothesis. That is, preserve the dependence in the data at two degrees when testing for third-order dependence. If the two degrees null is rejected, then one might test for four degrees against the null of three degrees (failing to reject such a test would lend further support to the notion that three degrees of separation is the limit of influence). Such a procedure constitutes a more powerful test than the permutation test with a null hypothesis that assumes no clustering at all.

Because the preceding limitation of the permutation test may not be widely recognized by either the social network or network science communities, the development of a test that used more realistic null hypotheses would be a valuable contribution. However, the test is not straightforward, and so this would constitute a worthy problem for statisticians. In the future, we hope that statisticians will make an increasing number of important contributions to this and other areas of social network analysis.

## Appendix

### Sources of bias in social influence analyses

The following are the major sources of bias discussed in this paper that may lead to misleading results in social influence analyses.

1. Latent homophily: Individuals form relationships because they have similar characteristics that continue to affect their outcomes post tie-formation. If the characteristics are unmeasured, their effects cannot be blocked and so will be indistinguishable from social influence (peer effects) 62.
2. Unmeasured common cause: An external factor that affects the outcomes of a group of individuals. If the external factor is not observed, then it will appear as though the change in the outcome of one individual tracks that of other individuals and thus is absorbed in the estimated network influence effect. If there exist dyads for which there should be no inter-individual influence, then such dyads can be used as a control group whose estimated peer effect represents the net effect of any unmeasured common causes. Under homogeneity assumptions, a difference-in-difference estimation strategy then enables the pure social influence effect to be recovered. This approach is the basis of the directionality test of CF 63,1.
3. Multiple peers: If the individuals whose peer effect is of interest are both influenced by the same third individual, then failing to account for the effects of this individual exposes the estimated peer effect to confounding bias. Such confounding is alleviated by simultaneously accounting for the effects of all alters.
4. Unbounded network: If the network is defined by arbitrarily drawing a boundary around a subset of the individuals, then those individuals at the ‘edge’ of the network may be exposed to more outside influences than individuals nearer the ‘center’ of the network. Thus, this problem manifests as a special case of the preceding item 3.

### Challenges and problematic issues facing social network models of relational data

The challenging features of modeling the network itself and using it to test for social selection are as follows.

1. Incomplete ascertainment: Complete sociocentric data can be difficult to obtain especially for large networks. Failure to measure all relationships may lead to distortions in network statistics involving three or more individuals and latent variables representing an unmeasured attribute or position in an unobserved social space. The use of a limited name generator (as in the FHS) is a common reason for incomplete ascertainment of ties (unless all unnamed individuals are assumed to have null ties with the ego).
2. Not accounting for sampling design: In general, there is often a discrepancy between the model describing the network of relationships in the population and that implied for a sample of individuals drawn from the population. For example, although network-based sampling schemes such as link-tracing designs are incredibly useful for generating networks of hard-to-reach populations 64,65), individuals' sampling probabilities can be difficult to determine, thus making the generation of population estimates problematic. However, methods that account for the sampling design used to generate network data have recently been developed (e.g., 66).
3. Model not generative: The parameters of ERGMs and related models are specific to the observed network and thus are unable to be generalized to networks with different *N* (the model does not describe how the effects will vary with *N*).
4. Computational problems: Models for sociocentric data can be challenging to fit because of the size of the network and, in the case of ERGMs, degeneracy 36.

### Glossary of terms

1. Social network: A set of individuals and the ties (relationships) linking them.
2. Tie: A connection between two individuals in the network; in our case, a tie designates a friendship nomination.
  1. Two degrees of separation: Two individuals linked by a two-path (one intermediary individual) that are not directly connected.
  2. Three degrees of separation: Two individuals linked by a three-path (two intermediary individuals) and no shorter paths.
3. Dyad: A pair of individuals in a network. In a directed network, the state of a dyad is the status of the pair of ties between the constituent individuals.
4. Triad: A triple of individuals in a network.
5. Social influence: The effect of one individual on another.
6. Social selection (homophily): The tendency of individuals with similar traits to form relationships with one another.
7. Degree: The number of ties an individual has with other individuals in the network. Equals the cardinality of the union of sets of an individual's in-degree and out-degree ties.
  1. Expansiveness: The ‘out-degree’ or the number of ties originating from an individual.
  2. Popularity: The ‘in-degree’ or the number of ties directed at the individual.
8. Density: The overall tendency of ties to form in the network. An unadjusted descriptive statistic is given by the number of ties in the network divided by the total number of possible ties.
9. Closure: The tendency for network configurations to be closed.
  1. Reciprocity: The tendency for mutual ties to form or ties to be reciprocated in the network. This is the simplest form of closure.
  2. Transitivity: The tendency for a tie from individual A to individual B to form if ties from individual A to individual C and from individual C to individual B exist. Commonly stated as ‘a friend of a friend is a friend.’ Reduces to general triadic closure in an undirected network.
  3. Cycle: A path that returns to its origin without backtracking. For example, the ties A–B, B–C, and C–A form a three-cycle.
10. Clustering: The tendency of ties to cluster and form densely connected regions of the network.
11. Degeneracy: A problem encountered when fitting ERGMs with highly collinear network statistics. Sometimes arises because a network contains regions of high and low density that are not captured by ERGMs that assume homogeneous effects across the network.

### Statistical software for network analysis

The following is a list of statistical software for fitting models to social network data. The list is not exhaustive.

1. statnet (http://statnet.org) in R. Suite of packages for network analysis in R.
  1. social network analysis (SNA). Package including a range of functions for descriptive analysis of networks. The lnam function fits the network autoregressive model and variants such as the network autocorrelation model 8.
  2. ergm. Package for fitting ERGMs.
  3. latentnet. Package for fitting latent space and latent cluster models.
2. pnet (http://www.sna.unimelb.edu.au/pnet/pnet.html). Program for simulation and estimation of ERGMs.
3. stocnet (http://www.gmw.rug.nl/stocnet/StOCNET.htm). Software system for the statistical analysis of social networks. Includes programs for stochastic blockmodels, the p _2_ model, and siena.
  1. siena (http://www.stats.ox.ac.uk/snijders/siena/). Software package with particular emphasis on longitudinal analysis of relational data. Can be run directly, through stocnet, or from R. Also fits ERGMs to cross-sectional relational data and allows simultaneous modeling of multivariate relational data.

Most of the software is available for modeling relational data, the reverse problem to that considered by CF. Only the SNA package in the StatNet system is designed to fit network autoregressive and autocorrelation models. However, we can adapt statistical software for spatial data (particularly areal data) to analyze network influence data.

We may use regular statistical packages to replicate the regression analysis performed by CF. However, as mentioned in the text, we must amend the standard GEE calculation if applied to network data with two-sided simultaneous relationships.
